# Combined Peridynamics and Discrete Multiphysics to Study the Effects of Air Voids and Freeze-Thaw on the Mechanical Properties of Asphalt

**DOI:** 10.3390/ma14071579

**Published:** 2021-03-24

**Authors:** Danilo Sanfilippo, Bahman Ghiassi, Alessio Alexiadis, Alvaro Garcia Hernandez

**Affiliations:** 1 Department of Civil Engineering, University of Nottingham, Nottingham NG7 2RD, UK; danilo.sanfilippo@nottingham.ac.uk; 2 School of Chemical Engineering, University of Birmingham, Birmingham B15 2TT, UK

**Keywords:** asphalt, mathematical modelling, peridynamics, discrete multiphysics

## Abstract

This paper demonstrates the use of peridynamics and discrete multiphysics to assess micro crack formation and propagation in asphalt at low temperatures and under freezing conditions. Three scenarios are investigated: (a) asphalt without air voids under compressive load, (b) asphalt with air voids and (c) voids filled with freezing water. The first two are computed with Peridynamics, the third with peridynamics combined with discrete multiphysics. The results show that the presence of voids changes the way cracks propagate in the material. In asphalt without voids, cracks tend to propagate at the interface between the mastic and the aggregate. In the presence of voids, they ‘jump’ from one void to the closest void. Water expansion is modelled by coupling Peridynamics with repulsive forces in the context of Discrete Multiphysics. Freezing water expands against the voids’ internal surface, building tension in the material. A network of cracks forms in the asphalt, weakening its mechanical properties. The proposed methodology provides a computational tool for generating samples of ‘digital asphalt’ that can be tested to assess the asphalt properties under different operating conditions.

## 1. Introduction

Asphalt, a heterogeneous mixture of aggregates, fillers and asphalt binder, is one of the most used infrastructure material. Asphalt’s mechanical properties are influenced by the properties of its constituents, its internal structure and the loading and environmental conditions during its service life. Understanding the degradation of asphalt, such as rutting, ravelling, freezing, strength loss and fatigue cracking, is important for better design, manufacture and maintenance of roads. 

One of the major sources of deterioration of asphalt is cracking. The fracture process can be divided into two different stages [1,2]: crack initiation and propagation. Crack initiation occurs when the mechanical stress is higher than a given limit, and micro-cracks occur in the mastic [3]. Under continuous load, these micro-cracks coalesce into macro-cracks, which initiate the propagation phase that ultimately, leads to failure [1]. The growth of microcracks damages asphalt irreversibly and increases maintenance costs [4], and this is influenced by different factors such as temperature, loading level and rate, fatigue and mixture composition. 

Another cause of asphalt failure in cold regions is thermal cracking (at low temperatures), which may significantly reduce the durability of pavements [5,6]. This damage is especially severe when water is present in the asphalt pores due to its comparatively high thermal expansion. Under icing temperatures, the internal pore structure in an asphalt mixture may change following a three-stage process [7]: (i) water expansion, which causes damage, and expansion of the exiting pores; at −10 °C, for instance, water undergoes 15% volume expansion [8]; (ii) cracking and merging of the pores and (iii) creation of new voids [9]. This phenomenon is especially intense in asphalts with a porosity between 6% and 13%, which retain part of the pore water. Asphalts with <6% voids have close pores that prevent water penetration, while asphalts with >13% voids have large pores that do not retain water [10,11,12].

There are many studies, both theoretical and experimental, on asphalt degradation (e.g., [3,4,13,14,15,16]), but only a few experimental works dedicated to low temperatures and freeze-thawcycles [5,6,7,17,18,19]. Furthermore, the experimental tests cannot show the evolution of the damage inside the asphalt’s microstructure, which is critical for understanding the deterioration mechanisms. To the best of our knowledge, there are also no available numerical methods for predicting degradation of asphalt or monitoring the damage progression resulting from freeze-thawactions. The development of such modelling tools, the subject of this paper, allows estimation of asphalt’s service life performance under cold environmental conditions. This is critical for optimising asphalt or developing novel materials with enhanced durability and long-term performance. 

Recent advancements in computer technology allow performing realistic simulations of the performance of materials across scales. Novel mesh-free methods are more suited for this purpose as they allow simulation of crack propagation and branching without the need for mesh regeneration. One of the simplest mesh-free methods is the lattice spring model (LSM) that divides solids into computational particles linked together with springs [20,21,22]. Peridynamics (PD) [23] is a novel mesh-free method developed as an improvement of the LSM [24] to allow better simulation of the material damage response. The application of PD to the simulation of damage in construction materials is highly innovative and limited, and its asphalt application will be presented in this paper for the first time.

This paper aims to develop a computational tool that allows realistic simulation of the damage of asphalt under mechanical and freeze-thawloads. We present a PD model coupled with discrete multiphysics developed in the LAMMPS molecular dynamics simulation package. This requires developing realistic models considering the aggregates and binder, voids and water, both in liquid and solid forms. Optical and micro-CT images are used to develop models considering the internal microstructure of a range of asphalt materials. PD implemented in LAMMPS also allows considering the plastic [25] and viscoelastic [26] response of materials and is therefore suitable for simulation of the response of asphalt materials under higher temperatures. The state-based Peridynamics is used here to evaluate the mechanical response of intact asphalt before and after being subjected to a freeze-thawcycle. The internal damage in the asphalt due to freezing is simulated by coupling Peridynamics with repulsive forces obtained from expansion of liquid phases and their transformation into solid phase using Discrete Multiphysics (DMP) [27,28,29]. The model is then used to discuss the effect of the freezing of the water present in the voids on the asphalt’s mechanical response.

## 2. Theory

### 2.1. Peridynamics

In Peridynamics, the material’s body is defined as a lattice, and contrary to classical continuum mechanics, its behaviour is defined through a constitutive equation that links deformation and force rather than strain and stress. The original bond-based Peridynamic approach [23] was limited to materials with a Poisson ratio ¼ in 3D and ⅓ in 2D. State-based Peridynamics [30] was introduced to overcome these limitations. In state-based Peridynamics, the forces that connect two bonded elements depend on the overall state of all the particles located within a material horizon rather than the single bond. The acceleration of any particle at position x in the reference configuration at time t is found from:

(1) $$\rho \left(x\right)ü\left(x,t\right)=\;\int_{H_{x}}\left\{\underset{-}{T}\left(x,t\right)\langle x^{\prime }-x\rangle -\underset{-}{T}\left(x^{\prime },t\right)\langle x-x^{\prime }\rangle \right\}{\mathrm{dV}}^{\prime }+b\left(x,t\right)$$

where ρ(**x**) is the mass density at **x**, **u** is the displacement vector field, H_x_ is a neighborhood of x with radius δ containing all the points $x^{\prime }$ within the horizon, $\underset{-}{T}\left(x,t\right)\langle x^{\prime }-x\rangle$ is the pairwise force state function at time *t* whose value is the force vector (per unit volume squared) acting between two particles within the horizon applied to the bond $\langle x^{\prime }-x\rangle$, ${\mathrm{dV}}^{\prime }$ is the infinitesimal volume, **b** is a body force density field. The relative position vector state of these two particles in the reference configuration **ε** is given by

(2) $$\underline{X}\langle x^{\prime }-x\rangle =x^{\prime }-x\;=\varepsilon$$

where **X** represents the reference state, mapping all bonds in a non-deformed body, B_0_. The relative displacement vector state **U** is associated with the bond by:

(3) $$\underline{U}\langle x^{\prime }-x\rangle =u\left(x^{\prime },t\right)-u\left(x,t\right)=\;\eta$$

**Figure 1:**
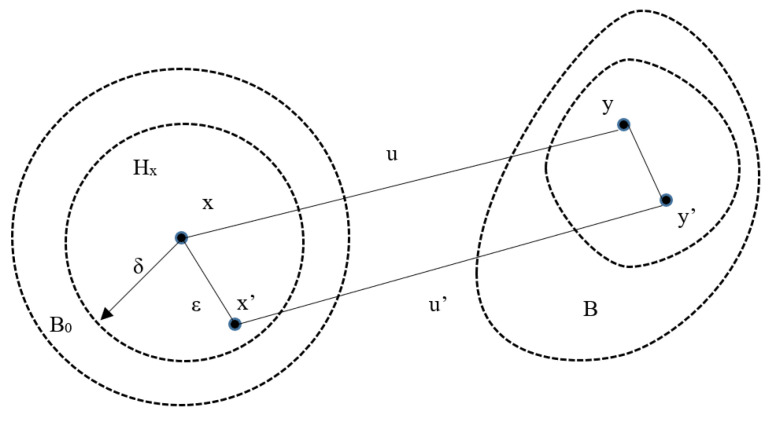
Deformation of the bond involved in (4) and in relation to the reference state ε, the deformation state η and the displacement state U.

The deformation state **Y** expressed in Equation (4) maps all bonds into its deformed image, **B**, Figure 1.

(4) $$\underline{Y}=\underline{X}+\underline{U}=\;y^{\prime }-y=\varepsilon +\eta$$

Material behaviour is modelled by spring-like bonds between particles **x** and **x’** within the horizon. The force acting on particles **x** and **x’** is:

(5) $$\underset{-}{T}\langle x^{\prime }-x\rangle =\underset{-}{f}\langle x^{\prime }-x\rangle \frac{\varepsilon +\eta }{||\varepsilon +\eta ||}$$

where f is the scalar part of the force state named the force modulus state.

The force scalar state is given by [25,31]:

(6) $$\underset{-}{f}=\;\frac{3K\theta }{m}\underset{-}{\omega }\underset{-}{x}+\alpha \underset{-}{\omega }\;\underline{e^{d}}$$

where ω is a scalar weighting function called the influence function whose argument is the bond vector **ε** in the reference configuration [31], e is the extension scalar state defined as $\underset{-}{e}=||\underline{Y}||-||\underline{X}||$, x is the reference position scalar state defined as $\underset{-}{x}\langle \varepsilon \rangle =||\varepsilon ||$, m is the weighted volume defined as m = (ωx)∙x, θ is the scalar state volume dilatation of the neighbourhood H defined as $\theta =\frac{3}{m}\left(\underset{-}{\omega }\underset{-}{x}\right)\underset{-}{e}$, e^d^ is the scalar deviator state component of the bond elongation defined as $\underline{e^{d}}=\underset{-}{e}-\frac{\theta ||X||}{3}$, *K* is the bulk modulus and α is related to the shear modulus *G* as:

(7) $$\alpha =\;\frac{15\;G}{m}$$

To model fracture, we introduce the notion of bond failure in relation to the bond’s stretch s defined by:

(8) $$s=\frac{||\underline{Y}\langle x^{\prime }-x\rangle ||-||\underline{X}\langle x^{\prime }-x\rangle ||}{||\underline{X}\langle x^{\prime }-x\rangle ||}=\;\frac{||\eta +\varepsilon ||-||\varepsilon ||}{||\varepsilon ||}$$

The breakage rule is that when *s* is larger than a critical value s_0,_ the bond breaks and is removed from the body. The critical value s_0_ is defined as [32]:

(9) $$s_{0}=\;\sqrt{\frac{5\pi \;G_{0}}{9\;K\;\delta }}$$

where *G*_0_ is the fracture energy. The value of *s*_0_ is not constant, but varies during the simulations based on its damage defined as:

(10) $$\varphi \left(x,t\right)=\;\frac{1-\;\int_{H_{x}}^{\;}\mu \left(x,t,\varepsilon \right){\mathrm{dV}}_{\varepsilon }\;}{\int_{H_{x}}^{\;}{\mathrm{dV}}_{\varepsilon }}$$

where μ is a history dependent damage function that takes on a value of 0 or 1, i.e.,:

(11) $$\mu \left(t,\varepsilon \right)=\;\{\begin{array}{c} 1\;\mathrm{if}\;s\left(t^{\prime },\varepsilon \right)<\;s_{0}\;\;\forall \;0\leq t^{\prime }\leq t\\ 0\;\;\;\;\;\;\;\;\;\;\;\;\;\;\;\;\;\;\;\;\;\;\;\;\;\;\;\;\;\;\;\;\;\;\;\mathrm{otherwise} \end{array}$$

For materials such as mastic, s_0_ depends on s_min_, the current minimum stretch among all bonds connected to a given material point [30]:

(12) $$s_{0}\left(t\right)=s_{00}-{\;\alpha s}_{\min}\left(t\right)$$

where s_00_ is a constant and:

(13) $$s_{\min}\left(t\right)=\;\min\;\left\{\frac{||\underset{-}{Y}\langle x^{\prime }-x\rangle ||\left(t\right)-||\underset{-}{X}\langle x^{\prime }-x\rangle ||}{||\underset{-}{X}\langle x^{\prime }-x\rangle ||}\right\}=\;\frac{||\eta +\varepsilon ||\left(t\right)-||\varepsilon ||}{||\varepsilon ||}$$

### 2.2. Modelling of Ice 

**Figure 2:**
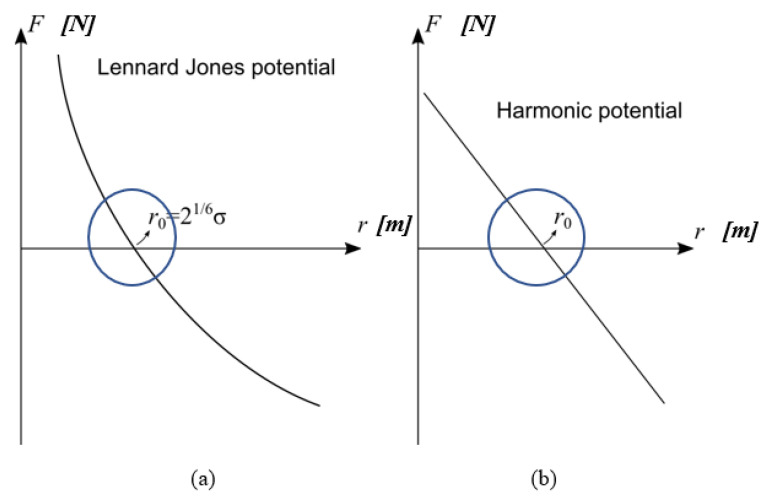
(**a**) Lennard Jones potential and (**b**) Harmonic potential.

To model ice, we take advantage of the flexibility of particle methods that can easily combine with other particle-based potentials in the context of discrete multiphysics. In this way, it is possible to extend the range of application of a single method by introducing potentials typical of other particle methods. This technique has been successfully used in several fields including fluid–structure interactions [33,34,35], solidification/dissolution [36,37], biological flows [38,39,40] and even machine learning [41,42]. In the case under investigation, we do not model water as a fluid. We are only interested in the pressure that expanding (i.e., freezing) water exerts on the asphalt structure. This can be achieved by a repulsion potential between water-water and water-asphalt particles. We use the positive (i.e., repulsive) branch of the Lennard Jones potential:

(14) $$U_{LJ}=4\varepsilon_{LJ}\left[{\left(\frac{\sigma_{LJ}}{r}\right)}^{12}-{\left(\frac{\sigma_{LJ}}{r}\right)}^{6}\right]\;\;\;r<2^{\frac{1}{6}}\sigma_{LJ}$$

where *r* is the distance between two particles, $\varepsilon_{LJ}$ is an energy constant that determines the particle’s rigidity and $\sigma_{LJ}$ is the distance at which the inter-particle potential is zero. The condition *r* < 2^⅙^$\sigma_{LJ}$ assures that only the repulsive part of the potential is used. Equation (14) comes from molecular dynamics, but here it provides a repulsive potential that avoids compenetration of water particles with mastic and aggregate particles (and among themselves) for distances smaller than $\sigma_{LJ}$. The value of *ε_LJ_* for ice can be approximated as follows (for simplicity, in the following discussion, we will name *ε_LJ_* and *σ_LJ_* simply as *ε* and *σ*). We assume that under the hypothesis of small deformations *r* ≈ *r*_0,_ the Lennard Jones potential approximates the Harmonic potential; see Figure 2. From the potentials in Equation (15), the forces are derived in Equation (16), Table 1.

Assuming that repulsive force between particles can be modelled as linear springs, the spring constant *k* is a function of the depth of the potential:

(17) $$F_{H}≅\frac{dF_{LJ}}{dr}\Delta r\to k≅-{\frac{dF_{LJ}}{dr}|}_{r_{0}}-\;\frac{dF_{LJ}}{dr}\;=\frac{12\varepsilon }{r_{0}^{2}}\left[13{\left(\frac{r_{0}}{r}\right)}^{14}-7{\left(\frac{r_{0}}{r}\right)}^{8}\right]$$

(18) $$k≅-{\frac{dF_{LJ}}{dr}|}_{r_{0}}=\frac{72\varepsilon }{r_{0}^{2}}$$

According to Kot et al. [43], the bulk modulus for a regular cubic lattice is:

(19) $$K=\frac{5}{3}\frac{k}{r_{0}}$$

and, therefore,

(20) $$\varepsilon ≅\frac{Kr_{0}^{3}}{120}$$

where ε is an energy constant, *K* is the bulk modulus and *r*_0_ is the lattice constant at which the potential is zero (i.e., *r*_0_ = *l*, the initial distance between water particles). Expansion is simulated by increasing the parameter *σ_LJ_*. During the simulation, *σ_LJ_* goes from *σ*^0^*_LJ_* = *l* [m] at *t* = 0 to *σ^END^_LJ_* = 1.05*l*, corresponding to a 15% volume expansion of water at the end of the simulation.

## 3. Methodology

The real and artificial asphalt mixtures used in this study are presented and discussed in this section. Both imaging of the section and micro-CT scan results are used to generate the initial microstructure of the asphalt models to evaluate the accuracy of the techniques used. The digital microstructures are then modified to represent a range of void % and saturation degree in the asphalt microstructure. The details of the processes followed are also presented in this section.

The numerical models, after validation, were used to simulate compressive tests on asphalt before and after being subjected to a cycle of freeze-thaw. The damage progression under compressive loading and the freeze-thaw was evaluated and discussed with the aim of the numerical results obtained. The details of the numerical analyses and the input parameters used are also presented in this section.

### 3.1. Mixtures 

The asphalt models used in the simulations were derived from samples of four types of asphalts: Dense Asphalt (DA), Porous Asphalt #1 (PA #1), Porous Asphalt #2 (PA #2) and Porous Asphalt #3 (PA #3), with target air voids of 5 10, 13 and 21%, respectively. The physical samples were prepared in the NTEC laboratories at the University of Nottingham, Nottingham, UK. CT-scanned and (as explained later) digitalised in a format readable by the software used to carry out the simulations. 

The composition, aggregate gradation and binder contents in the samples are shown in Table 2. For all mixtures, crushed limestone aggregates with a maximum size of 20 mm and 50/70 pen bitumen were used. The standards BS EN 13043:2013 for DA, BS EN 13108-1 for PA and BS EN 12697–33 were followed to manufacture the materials [44]. The materials were mixed at 160 °C and roller compacted at 140 °C. Asphalt slabs of 300 × 300 × 50 mm^3^ were produced. From the DA slab, a 35 × 35 × 55 mm^3^ was cut. From the slabs made of PA #1, PA #2 and PA #3, cores of 100 mm diameter and 50 mm height were extracted.

### 3.2. DA Model

**Figure 3:**
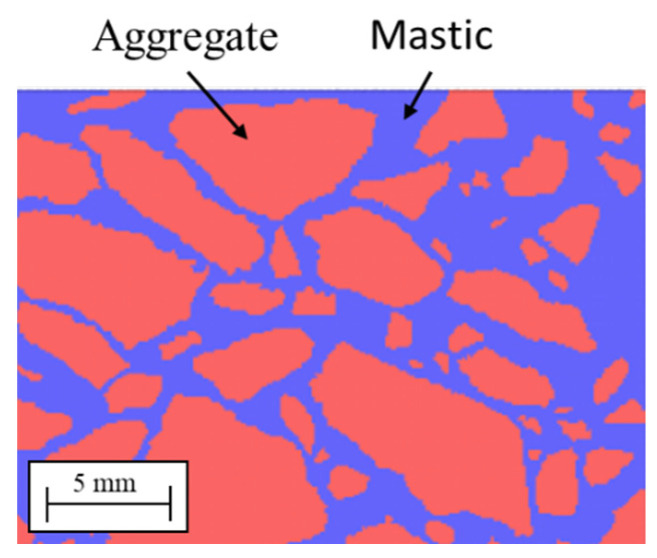
Asphalt (DA): blue particles represent the mortar; red particles represent the aggregate.

The DA sample surface was photographed using a digital camera with resolution 1257 × 896 and the pictures converted in a black and white image with MATLAB R2020a (The Math Works, Inc., Natick, MA, USA) and over imposed on a square lattice with side *l* = 10^−4^ m. Each node of the lattice corresponds to a Peridynamic particle: blue particles were created to represent mastic and red particles to represent aggregates, see Figure 3. According to the reference [45], aggregates greater than 1.18 mm can be considered part of the solid skeleton structure. Hence, mastic was defined as a mixture of aggregates ≤ 1.18 mm and bitumen. While we are aware that this is a simplification, we will assume that this value remains constant for the mixtures that we studied. 

In DA, voids were not considered due to the difficulty of identifying them using digital photography. DA has 190,000 particles for the mastic and 300,000 for the aggregates.

### 3.3. PA Models

A Phoenix v|tome|x L 300 micro CT scanner was used to scan PA asphalt samples under dry conditions; the X-ray tube was MXR320HP/11 (3.0 mm Be + 2 mm Al) from GE Sensing and Inspection Technology (Shanghai, China) operating with an acceleration voltage of 290 kV and a current of 1300 mA. 

We carried out the X-ray CT scans in the micro-computed tomography Hounsfield facility at the University of Nottingham, Nottingham, UK. We mounted the samples on a rotational table at a distance of 906.84 mm from the X-ray source. The reconstruction of scans was performed using GE Datos|x reconstruction software with 2× resolution to obtain a spatial resolution of 45.2 mm; the scans had an isotropic resolution, meaning that the slice thickness was also 45.2 mm. The raw images were 16-bit images, and the voxel value represented the x-ray attenuation. 

Then, ImageJ version 1.49 was used to process the images [46], convert them to 8-bit grayscale resolution and denoise the images to remove small clusters of voids and grains. The different material components such as aggregates, bitumen and air voids were extracted by segmenting the images based on grayscale thresholding using ImageJ version 1.49 (Rasband, W.S., ImageJ, U. S. National Institutes of Health, Bethesda, MD, USA).

The picture was over imposed on a square lattice with side *l* = 4 × 10^−4^ m using Matlab 2020a. As in the case of DA, each node of the lattice corresponds to a peridynamic particle: blue particles are assigned to mastic, and red particles are used to represent aggregates, see Figure 3. No computational particle was created in areas corresponding to the voids. Since the void fraction and aggregate size differed in the three samples, the number of particles was not the same. Sample PA #1 had 341,000 particles for the mortar and 367,000 for the aggregates; PA #2 148,000 particles for the mortar and 538,500 for the aggregates; PA #3 172,000 particles for the mortar and 455,000 particles for the aggregates.

### 3.4. Additional Asphalt Geometries 

**Figure 4:**
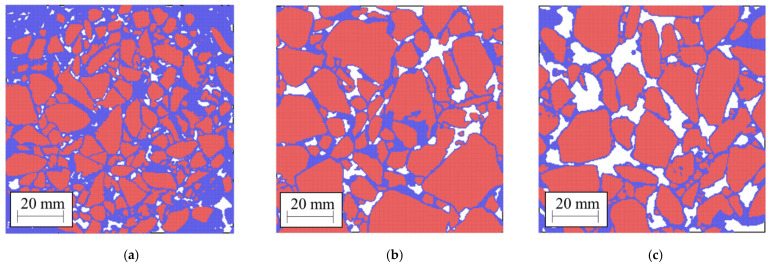
Samples (**a**) PA #1; (**b**) PA #2; (**c**) PA #3. Blue particles represent the mortar, red particles the aggregate.

**Figure 5:**
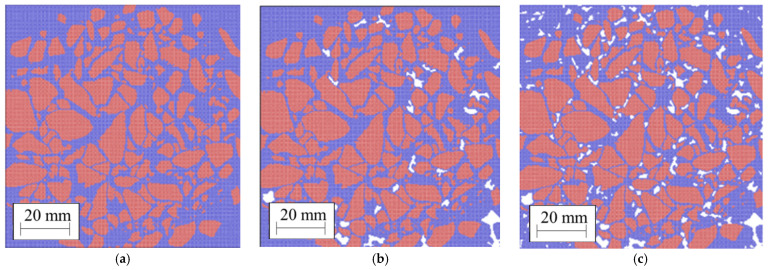
Examples of PA #1 with a range of air void contents. (**a**) 0%; (**b**) 5 %; and (**c**) 10 %.

To generate new geometries of asphalt mixtures with a range of air void properties, using ImageJ, we assumed that the mixtures from Figure 4 were the reference. From each of these specimens, we produced five different materials. (i) Without air voids; (ii) with the 25% smallest air voids; (iii) with the 50% smallest air voids; (iv) with 75% of the smallest air voids and (v) with 100% of the air voids (equivalent to the reference sample). See an example in Figure 5. The air voids’ geometries, including the average void area, diameter, perimeter, circularity and aspect ratio, were measured using the Particle Analysis function in ImageJ [45]. Finally, a suffix indicating the final void fraction was assigned to each generated sample. For example, PA #1/2.5% means that we started from PA #1 and filled all the voids so that the final void fraction was 2.5%. The aggregate gradation and binder contents are shown in Table 3. Increasing the amount of mastic, we add bitumen and dust smaller than 1.18 mm, keeping the skeleton structure constant.

Table 4 shows the topological properties of air voids in asphalt mixtures produced in this section. Similar results were presented in [12]. These results will be used below to evaluate the influence of freezing on the degradation of pavements.

### 3.5. Freeze-Thaw Simulation 

**Figure 6:**
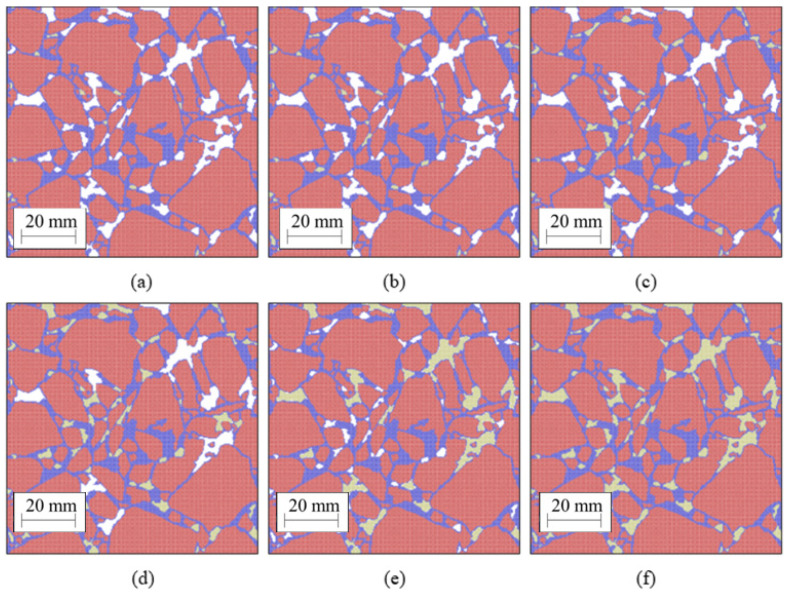
(**a**) PA #2/0.65% ice; (**b**) PA #2/1.3% ice; (**c**) PA #2/3.25% ice; (**d**) PA #2/6.5% ice; (**e**) PA #2/9.75% ice; (**f**) PA #2/13% ice Ice is represented by yellow particles.

We only used PA #2, which has a 13% air void content, to evaluate the effect of freeze-thaw on mechanical properties. For this purpose, we artificially filled some of the voids with ice, presented as yellow particles in Figure 6. To distinguish among samples, a suffix indicating the final ice content was assigned to each generated sample. For example, PA #2/0.65% means that we started from PA #2 and filled all the voids so that the final ice content was 0.65%. Figure 6 shows how this process was carried out. We started with the real PA #2 sample whose void fraction was 13%. Then, we gradually covered some of the voids (chosen randomly) with ice (yellow particles) until the ice content was 0.65%, Figure 6a, 1.3%, Figure 6b, 3.25%, Figure 6c, 6.5%, Figure 6d, 9.75%, Figure 6e and finally 13%, Figure 6f. The freeze-thaw simulation was performed following these steps:

- Water expands in the voids simulating ice formation, leading to cracking.
- After the water expansion is completed, the simulation is carried out for additional 10^6^ time steps to relax the system with no external load.
- Water shrinks in the void, simulating ice melting.
- Water is removed.
- After the water is removed, the simulation is carried out for additional 10^6^ time steps to relax the system with no external load.
- Finally, the sample is tested under simulated compression to assess mechanical response changes after the sample is subjected to a freeze-thawcycle.

### 3.6. Numerical Modelling Details and Input Parameters 

The intrinsic properties of the mastic and aggregate were the same for all simulations. In this study, we focused on temperatures below −10 °C and, therefore, we used the Peridynamic model for brittle materials discussed before. The mechanical properties used in the simulations of bitumen and asphalt mixtures are reported in Table 5; they were obtained from [47] and [48]. The peridynamic parameters of the asphalt binder used for the simulations are listed in Table 6. 

The calculation of the temperature profile inside the sample would require a non-isothermal model (the reader can refer to [37] for modelling heat transfer and phase transition with particle methods). During solidification, water remains at 0 °C because of the latent heat. The scenario we have in mind is when the water has permeated into the asphalt and freezes. We assume that the external temperature is sufficiently low; as a first approximation, the average temperature of the asphalt sample is close to −10 °C.

To simulate a uniaxial compressive test, each sample is placed into the simulation box between two rigid walls (boundary conditions). The simulations are carried out under plane stress conditions. For the model, this implies that we take a parallel slice with a thickness larger than the horizon and impose the stress along the *z* direction equal to zero. The physical parameters at the interface were set to the physical parameters of the mastic. The upper wall moves downward at a controlled velocity, and the lower wall is fixed. Uniaxial compression test simulation is carried out along the y-direction at a compression rate of 0.001 m/s; (we verified that quasi-static conditions were achieved at 0.001 m/s), the other directions were set to be free to expand or shrink. The time step used in all simulations was 10^−8^ s. 

The Peridynamic stress was calculated from the total force per volume, acting through the first layer of particles in contact with the upper wall. The resultant force was obtained by multiplying the particle’s volume and the average force density of the top layer. The simulations were carried out with the Peridynamics package [49] in LAMMPS/stable_7Aug2019-foss-2019a (http://lammps.sandia.gov) [50].

## 4. Model Validation

We modelled the tensile strength of bitumen beams tested in [45] to validate the accuracy of the modelling strategy and its parameters. For this purpose, we produced 3D and thin plate (i.e., pseudo 2D with the thickness slightly larger than the horizon simulated under the plane stress condition) models of the bitumen with different resolutions (number of particles used for development of the model) and checked the sensitivity of the results to these parameters.

**Figure 7:**
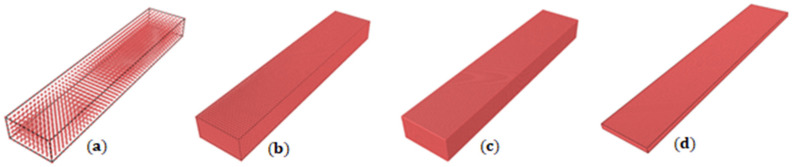
Geometries of bitumen for tensile tests. (**a**) 3D, l = 10^−3^ m; (**b**) 3D, l = 5 × 10^−4^ m; (**c**) 3D, l = 2 × 10^−4^ m; (**d**) example of a thin plate, l = 2 × 10^−4^ m.

The 3D specimen had dimensions of 1.0 × 5.0 × 0.5 cm^3^ simulated with four lattice resolutions in the range *l* = 10^−3^ – 10^−4^ m; see Figure 7a–c. In addition, the thin plate specimen had dimensions of 1.0 × 5.0 cm^2^ and a resolution of *l* = 10^−3^ – 10^−4^ m, see Figure 7d.

The number of particles in the 3D samples was 3366, 23,331, 332,826 and 2,580,641, for *l* 10^−3^, 5 × 10^−4^, 2 × 10^−4^ and 10^−4^ m, respectively. The number of particles in the thin plates was 3927, 14,847, 76,806 and 303,606, for *l* 10^−3^, 5 × 10^−4^, 2 × 10^−4^ and 10^−4^ m, respectively. The simulations were conducted at the two strain rates, 30 and 140 mm × min^−1^. The Peridynamic stress was calculated from the total force per volume, acting through the first layer of particles in contact with the upper wall.

**Figure 8:**
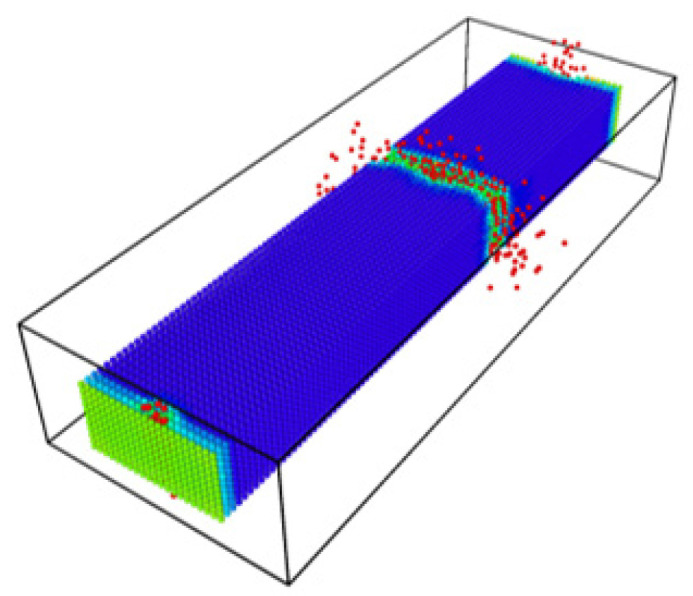
Broken bitumen sample in the simulation.

Figure 8 shows the bitumen’s beam’s failure in a 3D simulation showing that breakage is visually comparable with an equivalent experiment from the literature [45].

**Figure 9:**
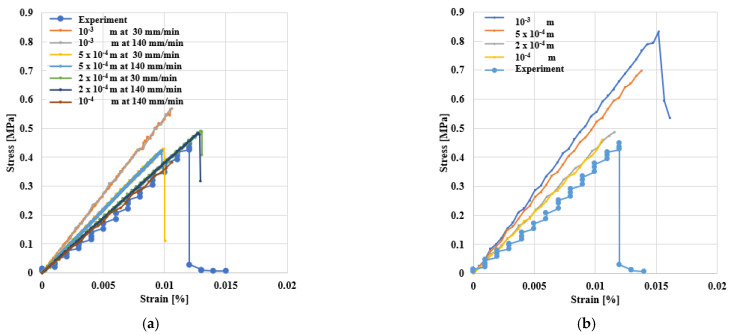
Stress/strain of bitumen beams at different resolutions and loading rate and comparison with other experiments [45]. (**a**) In 3D and (**b**) as a thin plate.

Figure 9a shows the tensile results of the 3D beam. Results are independent of the loading rate, and when the particle resolution is *l* < 2 × 10^−4^ m, simulations are very close to the experimental data. Figure 9b shows the 2D (thin plate) results. For *l* < 2 × 10^−4^ m, the results are independent of the particle resolution. However, contrary to the 3D results, they do not converge to the experimental data. In 2D, the greatest difference between the experiment and simulation is 10%. However, the 3D simulation at *l* = 10^−4^ m has eight times as many particles as the 2D simulation at the same resolution, which makes the simulation 16 times slower. Therefore, we decided to accept the error and run the simulations for thin plates at lattice *l* = 10^−4^ m. 

## 5. Results and Discussion

### 5.1. Compressive Response of DA Asphalt

**Figure 10:**
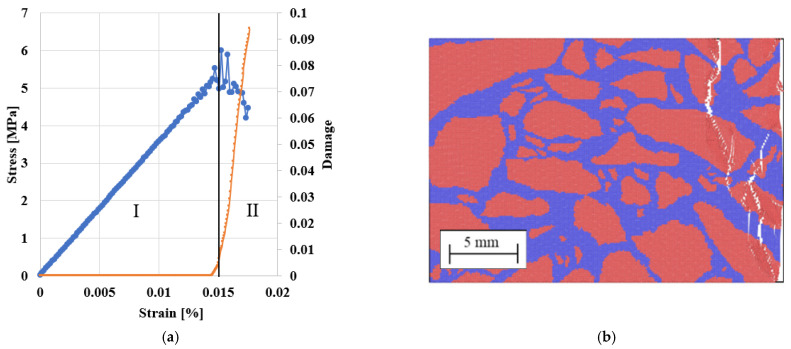
(**a**) Stress (blue curve) and the fraction of broken bond (damage, orange curve) versus strain. (**b**) Micro-crack formation in an asphalt specimen.

The result for the uniaxial compression test for each sample is shown Figure 10a. The stress-strain curve follows an equivalent tendency to that of experimental curves reported in the literature for asphalt at low temperatures showing brittle behaviour [51]. As expected, the stress increases with the compression, undergoing a sudden fracture and leading to total failure of the samples. 

The stress-strain curve and the percentage of broken Peridynamic bonds (damage) in the sample are compared in Figure 10a to illustrate the relationship between stress and damage. Two stages from the stress-strain curve can be distinguished during the failure process [52]. In Stage I (strain < 0.015%), there are no obvious cracks. Stage II occurs when the local strain reaches the critical value s_0_. Some of the bonds begin to break, generating micro-cracks in the aggregate. As the strain increases further, micro-cracks propagate, weakening the material, and the load exceeds the ultimate strength of the sample; micro-cracks evolve around the aggregates, resulting in large deformations and ultimately the destruction of the sample. Finally, Figure 10b shows that micro-cracks form mainly in the aggregate, especially at the interfaces, which can be considered the asphalt’s weak part. 

### 5.2. Compressive Response of PA Asphalt

Uniaxial compression tests of the PA test specimens mentioned above were also simulated to determine whether the peridynamics could capture the air voids’ influence on the asphalt’s compression strength. All the samples were subjected to the same load and boundary conditions as the DA samples. Figure 10 compares stress/strain curves for PA #1, PA #2 and PA #3, in the range of the air void contents studied.

**Figure 11:**
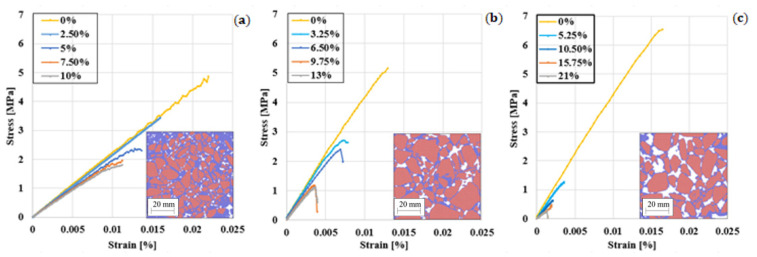
Stress/strain curves for PA #1 (**a**), PA #2 (**b**) and PA #3 (**c**) samples.

According to Figure 11, the asphalt stiffness and the peak load decrease by increasing the number of voids and their size. The maximal stress decreased by 64% (PA #1), 77% (PA #2) and 91% (PA #3) compared to the same asphalt with no voids, and the weakening of the material led to early breakage. In real asphalt, this could mean that the weakening of the material due to air voids’ presence leads to early breakage [2,30]. Hence, to produce durable asphalt, especially at lower temperatures, when the asphalt is prone to ravelling, it is advised that the content of mastic in the material is maximised. 

There are different types of asphalts that cover a wide range of compressive strengths depending on the bitumen, aggregate, fillers and voids. Our asphalt is in line with the compressive results for asphalts reported in the literature e.g. [53,54,55] at the same temperature. According to the sample and the void fraction, the model’s values in Figure 10 are between 0.5 and 3.5 MPa. Reference [53] reports values between 1.5 and 1.9 MPa, reference [54] between 2 and 3 MPa and [55] between 1.7 and 2.2 MPa, which are in the same range as our simulations. The model’s compressive results depend on the choice of parameters, and specifically G_0_, the fracture energy reported in Table 5, used in the simulations. G_0_ was taken from [48] and refers to weakly aggregated dolomite limestone [48], normally used to build road bases or binder courses. Hence, the compressive strength of asphalt will reflect the poor properties of these aggregates. Properties of additional aggregates can be found in reference [56].

Table 7 reports the Pearson’s correlation between the mechanical and the topological properties of the samples. The max stress and the max deformations are, respectively, the maximal stress and deformation before the sample’s failure, while for the equivalent Young’s Modulus, the slope of the linear part of the stress/strain curve, see Figure 11. 

As expected, a higher void content reduces the uniaxial compressive strength of the asphalt. Table 7 also shows that larger voids are more detrimental than smaller voids for the same void content. Moreover, given the same void size, samples with elongated and irregular shapes (i.e., high aspect ratio, low circularity) show, in general, lower ultimate strength and an equivalent Young’s modulus than samples with circular-like voids. The reasons for this are still unclear and will be investigated in future research. 

To compare changes of strength between the different types of asphalt analysed due to changes in gradation and amount of mastic, we have defined the parameter β as:

(21) $$\beta =\left(1-\frac{\mathrm{maximal}\;\mathrm{strength}\;\mathrm{of}\;\mathrm{the}\;\mathrm{asphalt}\;\mathrm{sample}}{\mathrm{maximal}\;\mathrm{strength}\;\mathrm{of}\;\mathrm{asphalt}\;\mathrm{with}\;0\%\;\mathrm{air}\;\mathrm{voids}}\right)\times 100$$

**Figure 12:**
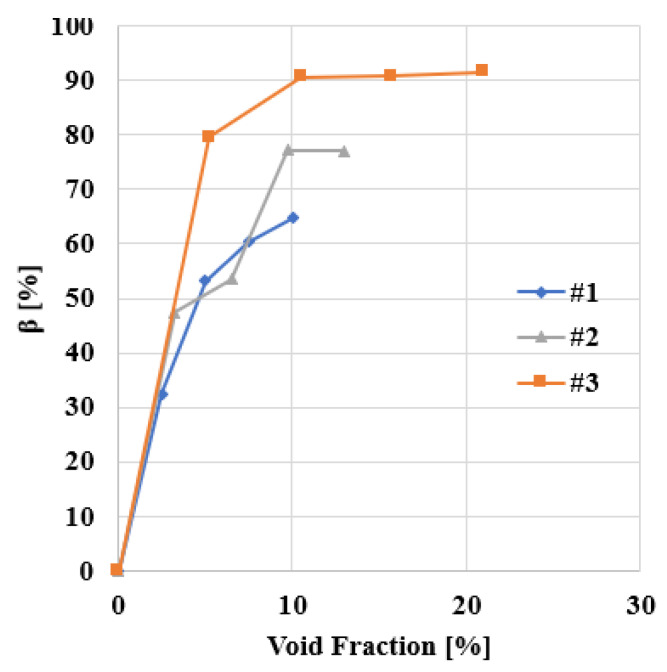
Changes of β with void %.

Finally, Figure 12 shows how *β* varies with the air voids fraction for PA #1, PA #2 and PA #3. It can be observed that small changes in the void fraction have a lower influence on the compressive strength of asphalt for densely packed mixtures. However, other types of mixtures, such as PA #3, could be extremely sensitive to changes in the amount of voids, for example, due to the lack of filler or changes in the source of dust, and extreme care should be taken during their design and manufacturing. This will be a point that we will analyse experimentally in future research.

### 5.3. Effect of Freeze-Thawon the Compressive Strength

**Figure 13:**
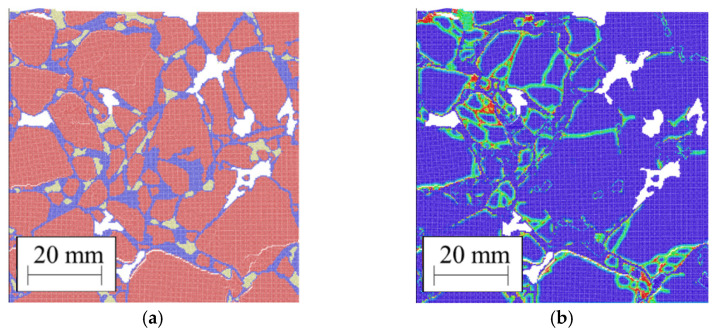
Cracking propagation due to ice expansion: (**a**) geometry; (**b**) damage.

Figure 13 shows the sample during the ice expansions. Breakage starts where ice expands and propagates at the bitumen-aggregate interface. This also creates new voids and increases the void fraction [7]. As expected, the freeze-thawcycle decreases the strength of asphalt [57,58,59]. To quantify this decrease and compare the simulations with experimental data, we define the reduction of the peak stress after freeze-thawas follows:

(22) $$\gamma =\left(1-\frac{\mathrm{peak}\;\mathrm{stress}\;\mathrm{of}\;\mathrm{the}\;\mathrm{sample}\;\mathrm{after}\;\mathrm{freeze}-\mathrm{thaw}}{\mathrm{peak}\;\mathrm{stress}\;\mathrm{of}\;\mathrm{the}\;\mathrm{intact}\;\mathrm{sample}}\right)\times 100$$

**Figure 14:**
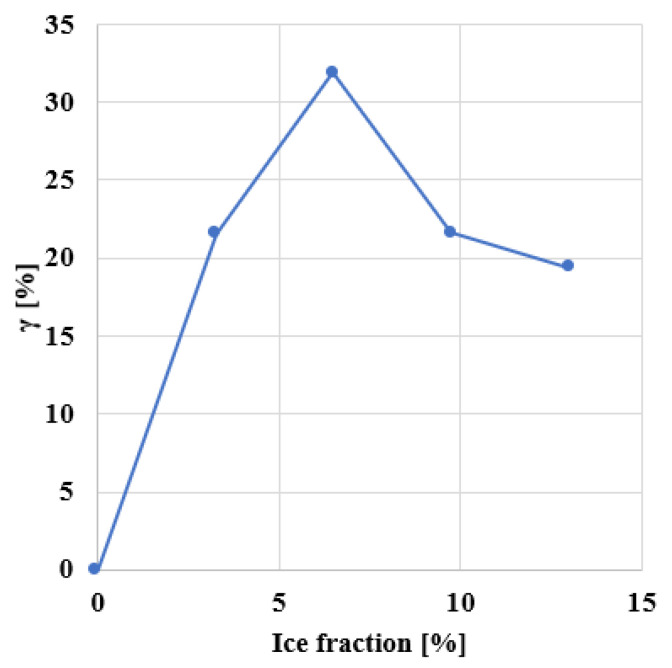
Reduction of peak stress with ice %.

Figure 14 shows how γ varies with the ice fraction and confirms the ice’s impact on fracture performance. During the ice expansion, cracks appear in the structure, and the stiffness of asphalt is compromised, leading to a reduction in the sample’s peak stress and earlier failure.

These results compare with experimental data. For example, reference [9] has determined that for a porous asphalt mixture with approximately 20% air void content, the strength loss can be higher than 40%. Further, most of the strength is lost after the first cycle, as shown in Figure 13. In future research, this computational framework will be used to better understand the influence of air voids’ geometrical properties on the resistance of the asphalt to freeze-thawcycles.

## 6. Conclusions

In this article, we have demonstrated the use of Peridynamics combined with Discrete Multiphysics to model crack formation and propagation in asphalt at low temperatures taking into account air voids and ice formation. Find below some of the conclusions:

- This paper demonstrates a way to understand how microcracks are formed in the asphalt under freezing conditions, a phenomenon that is extremely difficult to observe in experiments.
- The simulations show the model’s reliability in obtaining a mechanical response comparable with experimental tension and compression tests of bitumen and asphalt, respectively.
- As expected, the higher the void fraction, the higher the loss of compressive strength of an asphalt mixture. Further, the size and shape of the voids affect the strength of the asphalt. Larger voids are more detrimental than smaller voids, especially if they have a high aspect ratio and low circularity.
- Using this model, we observed that the amount of mastic in densely packed mixtures does not have a strong influence on the compressive strength of asphalt. However, less densely packed mixtures, such as porous asphalt, are more sensitive to the amount of mastic in the asphalt.
- The model can also assess the effect of ice formation on the asphalt structure. Water particles are created in the voids, and their volume is increased with time to simulate solidification. The simulations show the formation of cracks produced by water expansion during solidification and the consequent loss in mechanical strength. To the best of our knowledge, water expansion in cavities has not been simulated to date.

This methodological study provides researchers in the field with a powerful new tool for understanding the behaviour of asphalt under scenarios that, so far, have not been accessible to computer simulation. The systematic study of asphalt mechanical properties changes due to the size, number and distribution of ice-filled voids will be done in future research.

## Figures and Tables

**Table 1:** Lennard Jones and Harmonic potentials and forces.

Lennard Jones	Harmonic	
$\mathrm{Potential}:\;E_{LJ}=4\varepsilon \left[{\left(\frac{\sigma }{r}\right)}^{12}-{\left(\frac{\sigma }{r}\right)}^{6}\right]=\varepsilon \left[{\left(\frac{r_{0}}{r}\right)}^{12}-2{\left(\frac{r_{0}}{r}\right)}^{6}\right]$	$\mathrm{Potential}:\;E_{H}=\frac{1}{2}k\Delta r^{2}$	(15)
$\mathrm{Force}:\;F_{LJ}=\frac{12\varepsilon }{r_{0}}\left[{\left(\frac{r_{0}}{r}\right)}^{13}-2{\left(\frac{r_{0}}{r}\right)}^{7}\right]$	$\mathrm{Force}:\;F_{H}=-k\Delta r$	(16)

**Table 2:** Asphalt mixture composition.

Size (mm)	Passing (%) DA	Passing (%) PA #1	Passing (%) PA #2	Passing (%) PA #3
**20**	0.9	0.0	20	10
**14**	15.8	0.0	25	38
**10**	21.3	35.1	26	35
**6.3**	14.2	19.3	7	0
**Dust**	47.8	45.6	22	17
**Bitumen**	4.7	4.5	4.2	3.3
**Air void content**	5.0	10.0	13	21

**Table 3:** Asphalt mixture composition.

	Passing (%)											
Size (mm)	PA#1 7.5%	PA#1 5%	PA#1 2.5%	PA#1 0%	PA#2 9.75%	PA#2 6.5%	PA#2 3.25%	PA#2 0%	PA#3 15.75%	PA#3 10.5%	PA#2 5.25%	PA#3 0%
**20**	0.0	0.0	0.0	0.0	19.5	19.0	18.5	18.1	9.8	9.2	8.9	8.6
**14**	0.0	0.0	0.0	0.0	24.5	23.8	23.2	22.7	36.3	35.0	33.5	32.3
**10**	34.5	33.9	33.3	32.5	25.3	24.7	24.1	23.5	33.4	32.2	30.9	29.7
**6.3**	18.9	18.5	18.2	18.0	6.8	6.7	6.6	6.4	0.0	0.0	0.0	0.0
**Dust above 1.18 mm [46]**	30.0	29.4	28.8	28.4	8.8	8.6	8.4	8.3	6.7	6.4	6.2	6.0
**Dust below 1.18 mm [46]**	16.6	18.2	19.7	21.1	15.1	17.2	19.2	21.0	13.8	17.2	20.5	23.4
**Bitumen**	5.2	5.9	6.5	7.1	5.2	6.2	7.0	7.9	5.1	6.8	8.2	9.5
**Air void content**	7.5	5.0	2.5	0.0	9.75	6.5	3.25	0	15.75	10.5	5.25	0

**Table 4:** Topological properties of the voids calculated from the CT-scans.

Sample	Void Content [%]	Mean Void Diameter [mm]	Mean Void Area [mm^2^]	Mean Void Perimeter [mm]	Mean Void Aspect Ratio [—]	Mean Void Circularity [—]
**PA #1/0%**	0.00	0.00	0.00	0.00	0.00	0.00
**PA #1/2.5%**	2.50	1.19	1.11	2.80	1.87	0.72
**PA #1/5%**	5.00	1.47	1.70	3.63	1.99	0.67
**PA #1/7.5%**	7.50	1.67	2.19	4.27	2.03	0.65
**PA #1/10%**	10.00	1.82	2.60	4.82	2.03	0.64
**PA #2/0%**	0.00	0.00	0.00	0.00	0.00	0.00
**PA #2/3.25%**	3.25	2.22	3.87	7.83	2.41	0.54
**PA #2/6.5%**	6.50	3.29	8.50	10.05	2.41	0.51
**PA #2/9.75%**	9.75	3.82	11.46	11.60	2.38	0.49
**PA #2/13%**	13.00	3.42	9.18	13.48	2.40	0.48
**PA #3/0%**	0.00	0.00	0.00	0.00	0.00	0.00
**PA #3/5.25%**	5.25	3.73	10.92	20.75	2.29	0.48
**PA #3/10.5%**	10.50	4.79	18.01	23.80	2.11	0.49
**PA 3/15.75%**	15.75	5.57	24.35	23.95	2.16	0.47
**PA #3/21%**	21.00	6.06	28.83	19.36	2.11	0.51

**Table 5:** Mechanical properties of bitumen, mastic and aggregates at −10 °C used in the simulations.

Material	ρ [kg m^3^]	*E* [GPa]	ν [-]	G_0_ [kJ/m^2^]
**Bitumen, PG64-22 at −18 ℃** [45]	1000	3.7	0.30	—
**Mastic** [48]	2200	18.2	0.25	270.00
**Aggregates** [48]	2500	56.8	0.15	0.25
**Interface mastic/aggregates** [48]	—	18.2	0.25	77.00

**Table 6:** Peridynamic parameters used in the simulations. _s00_ is defined by Equation (9).

Model	*l* [m]	*s*_00_ [—]	α [—]	N
**Bitumen beams (3D)**	1 × 10^−3^	1.2 × 10^−4^	0.30	3366
	5 × 10^−4^	1.2 × 10^−4^	0.30	23,331
	2 × 10^−4^	1.2 × 10^−4^	0.30	332,826
	1 × 10^−4^	1.2 × 10^−4^	0.30	2,580,651
**Bitumen beams (Thin plate)**	1 × 10^−3^	2.0 × 10^−4^	0.30	3927
	5 × 10^−4^	2.0 × 10^−4^	0.30	14,847
	2 × 10^−4^	2.0 × 10^−4^	0.30	76,806
	1 × 10^−4^	2.0 × 10^−4^	0.30	303,606
**Mastic, DA**	1 × 10^−4^	6.4 × 10^−3^	0.25	—
**Aggregate, DA**	1 × 10^−4^	1.3 × 10^−4^	0.25	—
**Interface, DA**	1 × 10^−4^	3.4 × 10^−3^	0.25	—
**Mastic, PA**	4 × 10^−4^	3.2 × 10^−3^	0.25	—
**Aggregate, PA**	4 × 10^−4^	6.5 × 10^−5^	0.25	—
**Interface, PA**	4 × 10^−4^	1.7 × 10^−3^	0.25	—

**Table 7:** Pearson correlations between the mechanical and the topological properties of the samples.

Properties	Void Content	Mean Void Diameter [mm]	Mean Void Area	Mean Void Aspect Ratio	Mean Void Circularity
**Ultimate strength**	−0.62	−0.64	−0.58	−0.52	−0.37
**Ultimate strain**	−0.77	−0.92	−0.82	−0.73	−0.41
**Equivalent Young modulus**	−0.26	0.02	0.09	−0.32	−0.57

## Data Availability

The code used for the simulations is freely available under the GNU General Public License v3 and can be downloaded from the University of Birmingham repository http://edata.bham.ac.uk/568/.
